# Inhibition of lactylation by LRP1 expression increases the risk of intervertebral disc degeneration: A multi-omics summary-based Mendelian randomization analysis

**DOI:** 10.1097/MD.0000000000044795

**Published:** 2025-09-19

**Authors:** Yang Yang, Zhen Ai, Dingxuan Liu, Xi Gao

**Affiliations:** a Heilongjiang University of Chinese Medicine, Heilongjiang, China; b First Hospital of Heilongjiang University of Chinese Medicine, Heilongjiang, China.

**Keywords:** intervertebral disc degeneration, lactylation, LRP1, QTL, summary-based mendelian randomization

## Abstract

Intervertebral disc degeneration (IVDD) causes neck, back and low back pain, and is a major global public health problem. Lactate, a metabolic product of disc cells, relates closely to degeneration. Lactylation, driven by lactic acid, associates with IVDD; targeting related genes may offer new therapies. This study aimed to identify causal lactylation-related genes in IVDD via multi-omics summary-based Mendelian randomization (SMR). In this study, data from a genome-wide association study (GWAS) were combined with methylation quantitative trait loci (mQTL) and expression quantitative trait loci (eQTL) to investigate the relationship between lactylation-related genes and disc degeneration. Lactylation-related genes from GeneCards, IVDD GWAS data from FinnGen, cis-eQTL data from eQTLGen Consortium, cis-mQTL data from SMR. SMR analysis and HEIDI tests were used to assess causality, and associations within mQTL-eQTL pathways were analyzed using multicohort data. Significance of results was determined using p_SMR < .05 and p_HEIDI > .01. At the gene expression level, 11 lactylation-related genes were identified, of which KAT5, CEACAM6, NR6A1, MRE11, LUC7L2, H2BC12, and RARG were negatively correlated with the risk of IVDD, and H4C8, SIRT1, H2AC6, and LRP1 were positively correlated with the risk of IVDD. At the DNA methylation level, 27 CpG sites near 14 genes were found to have a causal effect on IVDD, however, when combined with the causal effect of gene expression, only 11 CpG sites near 4 genes existed that had a causal effect. After integrating the multi-omics data between mQTL and eQTL, we identified 3 lactylation-related genes, KAT5, CEACAM6, and LRP1. cg01515074 methylation-induced upregulation of LRP1 increases degeneration risk, while cg12146864 methylation-mediated downregulation of LRP1 reduces this risk, collectively reinforcing the role of LRP1 as a key driver in IVDD pathogenesis. In this study, we identified lactylation-related genes that have a causal role in IVDD, mainly LRP1. The resulting study emphasizes the importance of LRP1 in the pathogenesis of IVDD, with the potential to be a therapeutic target. Integration of multi-omics data has provided new understanding in the molecular mechanisms of IVDD pathogenesis and new strategies for targeted therapy.

## 1. Introduction

The intervertebral disc (IVD) is a fibrocartilaginous structure that connects 2 neighboring vertebral bodies, and consists of a peripheral ring of annulus fibrosus, a central nucleus pulposus (NP), cartilaginous endplates (CEP) on the upper and lower surfaces, and a large extracellular matrix (ECM). IVD can withstand strong compression, bending and torsion loads, has the ability to exhibit athleticism, weight-bearing, and flexible properties. It is also involved in the protection of neuroanatomically related structures throughout the spine.^[1]^ The human spine consists of 23 intervertebral discs, when intervertebral disc degeneration (IVDD) occurs, it usually results in changes in spinal anatomy, gradual onset of uncomfortable symptoms such as pain in the neck, back and lower back.^[2]^ Low back pain is the leading cause of disability worldwide and imposes a significant societal burden. IVDD as one of the most important reasons for the development of low back pain, has therefore been a hot topic of research.^[3]^

Although IVDD is one of the normal manifestations of aging, studies have found a trend toward younger IVDD, its etiology is complex and is usually closely related to a variety of injuries, such as IVD morphology exceptions, mechanical overloads. IVDD is characterized primarily by a degenerative cascade reaction associated with chronic inflammation, which is closely related to the metabolic homeostasis of the IVD.^[4,5]^ It is important to study targeted therapies related to IVDD metabolism.

IVD has a special anatomy, annulus fibrosus (AF) and EP wrap around the NP, forming the IVD into a closed, anaerobic, avascular tissue, thus, a healthy disc is an organ with special immune competence.^[6]^ Nutrition of the IVD relies primarily on cellular metabolism within the IVD, thus anaerobic glycolysis is the main mode of energy supply for IVD, with NP cells being the main energy donor cells.^[1,4,7,8]^

The end product of anaerobic glycolysis is lactate. Over time, this physiologic buildup of lactate over time the IVD are in an acidic environment. While the IVD adapts to this low pH, studies have found^[7,9]^ that high levels of lactate are a hallmark of IVDD and contribute to exacerbations, this may be related to the fact that lactate accumulation promotes apoptosis and autophagy in NPCs and facilitates the activation of inflammatory pathways, ultimately accelerating the cascading degenerative response of the IVD. In the past, lactate was considered a metabolic waste product, however, with increasing research, lactate was found to be involved in the lactylation process as a driving factor. Lactylation (Lac) is a post-translational modification of proteins, Lac occurs on lysine residues in proteins, both histones and non-histone proteins.^[10]^ It has been found^[9]^ that the occurrence of Lac in normal IVD or early IVDD tissue affects the energy metabolism of NPCs as well as the immune environment. As the degree of IVDD changes, macrophage infiltration occurs and NPCs adapt to the high-lactate environment, and Lac plays a role in promoting repair of degenerative disc disease. Lac is associated with specific genes and regulates gene transcription through epigenetic mechanisms,^[10]^ however, the genetic correlation between Lac and IVDD has not been clearly studied.

Summary-based Mendelian randomization (SMR) study is a method of causal inference based on genetic variation. SMR uses genetic variation as an instrumental variable to infer causal relationships between exposure and outcome, thereby revealing potential therapeutic targets. In addition, SMR integrates genomic, epigenomic, and other data to ultimately identify disease-causing genes associated with intervertebral disc degenerative disease (IVDD).

This study uses SMR to integrate IVDD genome-wide association study (GWAS) data with mQTL and eQTL of lactylation-related genes, aiming to clarify the link between lactylation and IVDD.^[11]^ Our key findings highlight LRP1 as a critical lactylation-related gene driving IVDD: its expression, regulated by the cg01515074 methylation site, increases IVDD risk by inhibiting lactylation. This fills a knowledge gap in understanding genetic and epigenetic links between lactylation and IVDD, offering novel insights into pathogenesis and targeted therapy development.

## 2. Methods

### 2.1. Study design

**Figure 1.**
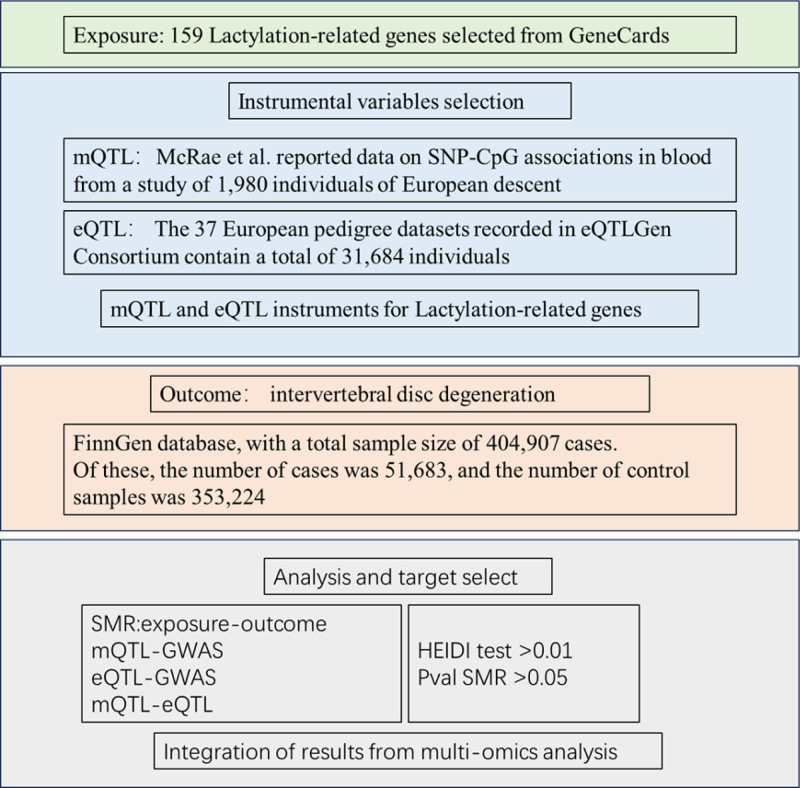
The overall design of this study. This figure illustrates the overall analytical framework of the study, including the following core steps: Data sources: 159 lactylation-related genes were selected from the GeneCards database as exposure factors; cis-eQTL data were obtained from the eQTLGen Consortium (37 datasets of European populations, totaling 31,684 individuals); cis-mQTL data were derived from the SMR database (SNP-CpG association data of peripheral blood from 1980 individuals of European ancestry); outcome data for IVDD were retrieved from the FinnGen database (404,907 samples, including 51,683 cases and 353,224 controls). Analysis workflow: A 3-step summary-based Mendelian randomization (SMR) approach was adopted to sequentially integrate eQTL and mQTL of lactylation-related genes with GWAS data of IVDD. The HEIDI test (p_HEIDI > .01) was used to exclude pleiotropy interference, and finally, genes and regulatory loci with causal associations with IVDD were screened out. Core objective: To clarify the potential mechanisms by which lactylation-related genes affect IVDD through expression regulation or methylation modification, and to provide candidate targets for targeted therapy. eQTL = expression quantitative trait loci, IVDD = intervertebral disc degeneration, mQTL = methylation quantitative trait loci, SNP = single nucleotide polymorphism.

Figure 1 illustrates the overall design of this study. Initially, we included 159 lactylation-related genes from the GeneCards database. Next, we combined Lac-related genes with eQTL data provided by the eQTLGen Consortium database, mQTL data provided by the SMR database by using the SMR approach, acquisition of Lac-eQTL, Lac-mQTL. Subsequently, by using a 3-step SMR approach, we combined GWAS for IVDD with Lac-eQTL, Lac-mQTL to obtain genes associated with IVDD.

### 2.2. Data sources

#### 2.2.1. Lactylation-related genes acquisition

Lactylation-related genes were extracted from the GeneCards database (https://www.genecards.org/) using the keyword “lactylation,” yielding 159 related genes. GeneCards integrates information from nearly 200 authoritative sources (e.g., NCBI, UniProt, OMIM) and provides multidimensional data on genomics, transcriptomics, proteomics, and clinical medicine. Compared with databases like GenBank and Ensembl, it features high integration, linking clinical data, drug information, and functional annotations to meet cross-field needs.

#### 2.2.2. QTL data

Cis-eQTLs are single nucleotide polymorphism (SNP) loci close to regulated genes, directly reflecting how genetic variation affects gene expression and offering insights into regulatory mechanisms. Cis-mQTLs refer to methylation sites near regulated genes; their methylation level changes can influence gene expression, providing direct evidence of epigenetic regulation and addressing the neglect of epigenetic factors in traditional genetic studies.

The cis-eQTL data were obtained from the eQTLGen Consortium (https://www.eqtlgen.org/phase1.html), including 37 datasets with 31,684 individuals of European descent.

Cis-mQTL data from SMR (https://yanglab.westlake.edu.cn/software/smr/#mQTLsummarydata) included 1980 individuals, with raw data from 2 peripheral blood cohorts: BSGS (n = 614) and LBC (n = 1366).^[12]^ Methylation status of European samples was measured using the Illumina HumanMethylation450 chip, and the pooled data were a meta-analysis of BSGS and LBC.^[13]^ Only DNA methylation probes with at least one cis-mQTL (*P* < 5e−8) and SNPs within 2Mb of each probe were included.

#### 2.2.3. GWAS data acquisition for intervertebral disc degeneration

IVDD GWAS data from FinnGen database (https://www.finngen.fi/en/access_results), with a total sample size of 404,907 (51,683 cases and 353,224 controls), all of European origin. IVDD diagnosis was based on the International Classification of diseases (ICD), specifically ICD-10 (M51), ICD-9 (722), and ICD-8 (725) coding standards.

## 3. Summary-data-based MR analysis

Compared to traditional MR analysis, the SMR approach utilizes cis-QTL and obtains higher statistical efficacy, especially when the exposure and outcome data come from 2 independent samples with large sample sizes. Top-ranked association cis QTL were selected by considering a window (±1000 kb) centered on each associated gene and meeting a *P*-value threshold of 5.0e−8. SNPs with allele frequency differences between any paired datasets exceeding a specified threshold (the default value is 0.2) were excluded, including LD reference samples (1000 Genomes European reference), QTL data, and outcome data. HEterogeneity in Dependent Instruments (HEIDI) test was used to distinguish between pleiotropy and chaining, with p_HEIDI < .01 indicating possible pleiotropy leading to exclusion from the analysis.

In this study, SMR analysis was used to assess the association between methylation and expression of Lac-related genes and IVDD. We constructed a hypothetical mediator model that suggests that individual SNPs influence traits by altering levels of DNA methylation (DNAm), which in turn regulates the expression levels of functional genes.^[14]^ In this study, we mainly analyzed by 3-step SMR, using SNP as a tool, Lac-eQTL as an exposure, and IVDD as an outcome; using SNP as a tool, Lac-DNAms as an exposure, and IVDD as an outcome; and using SNP as a tool, Lac-DNAms as an exposure, and Lac-eQTL as an outcome. The results of the SMR analyses were visualized by covariance scatter plots for visualization.

## 4. Statistical analysis

Final candidate gene inclusion criteria were defined as follows: genome-wide significance in eQTL, mQTL, and GWAS data (all with *P* < .05); no significant heterogeneity in HEIDI test results (p_HEIDI > .01) to exclude potential pleiotropy; and consistent causal effects across the 3-step SMR analysis, ensuring the robustness of associations with IVDD.

The SMR analysis and HEIDI tests were performed using SMR software (version 1.3.1) (https://yanglab.westlake.edu.cn/software/smr/#ExecutableFiles(version1.3.1), following the tool’s standard workflow for causal inference. Covariance scatterplots for visualizing SMR results (e.g., associations between gene expression/methylation and IVDD) were generated using R software (version 4.4.2) with the “magick” and “TeachingDemos” packages, and the “plot_SMR.r” script (https://yanglab.westlake.edu.cn/software/smr/#RscriptforSMRlocusplot) to illustrate effect sizes and significance.

## 5. Results

**Figure 2.**
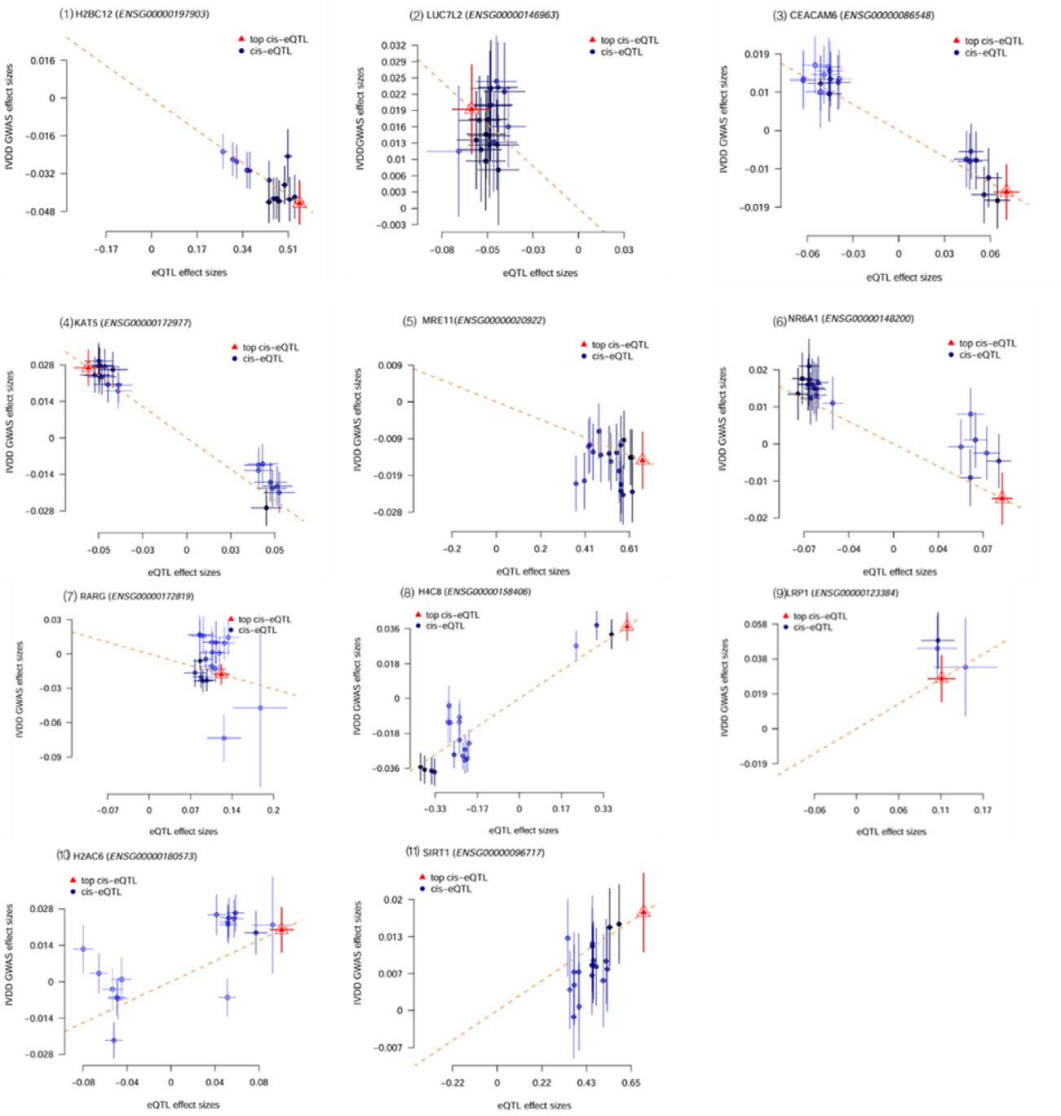
The effect of Lac-related genes expression on IVDD. This figure visualizes the expression effects of 11 lactylation-related genes significantly associated with IVDD in the first step of SMR analysis (p_SMR < .05, p_HEIDI > .01). Each subplot corresponds to 1 gene, with the horizontal axis representing eQTL effect sizes and the vertical axis representing GWAS effect sizes. The core results are as follows: Negatively correlated genes (7): KAT5, CEACAM6, NR6A1, MRE11, LUC7L2, H2BC12, RARG, suggesting that high expression of these genes may reduce the risk of IVDD. Positively correlated genes (4): H4C8, SIRT1, H2AC6, LRP1. Among them, the positive correlation of LRP1 is particularly significant (topSNP: rs55909821), indicating that its high expression may increase the risk of IVDD. IVDD = intervertebral disc degeneration.

Table 1 shows the results of the first step of the SMR analysis of the effect of Lac-related genes expression on IVDD. A total of 11 gene expressions were correlated with IVDD (p_SMR < .05, p_HEIDI > .01). Among these, 7 genes showed a negative correlation with IVDD risk, meaning higher expression of these genes was associated with a lower risk of IVDD. In contrast, 4 genes were positively correlated with IVDD risk, indicating that their increased expression was linked to a higher risk of the disease. These associations were further validated by visualization, which confirmed the directionality of the effects (Fig. 2).

**Table 1:** The effect of Lac-related genes expression on IVDD.

	ProbeID	Gene	topSNP	p_SMR	p_HEIDI
1	ENSG00000158406	H4C8	rs3999544	.0000	.0515
2	ENSG00000197903	H2BC12	rs12192049	.0000	.0143
3	ENSG00000172977	KAT5	rs502468	.0006	.4570
4	ENSG00000096717	SIRT1	rs10997832	.0126	.6680
5	ENSG00000180573	H2AC6	rs4645	.0239	.0207
6	ENSG00000086548	CEACAM6	rs6508997	.0295	.9050
7	ENSG00000148200	NR6A1	rs10122154	.0376	.0784
8	ENSG00000020922	MRE11	rs535801	.0383	.1460
9	ENSG00000146963	LUC7L2	rs6962053	.0389	.3800
10	ENSG00000123384	LRP1	rs55909821	.0400	.1670
11	ENSG00000172819	RARG	rs7969717	.0471	.0414

**Figure 3.**
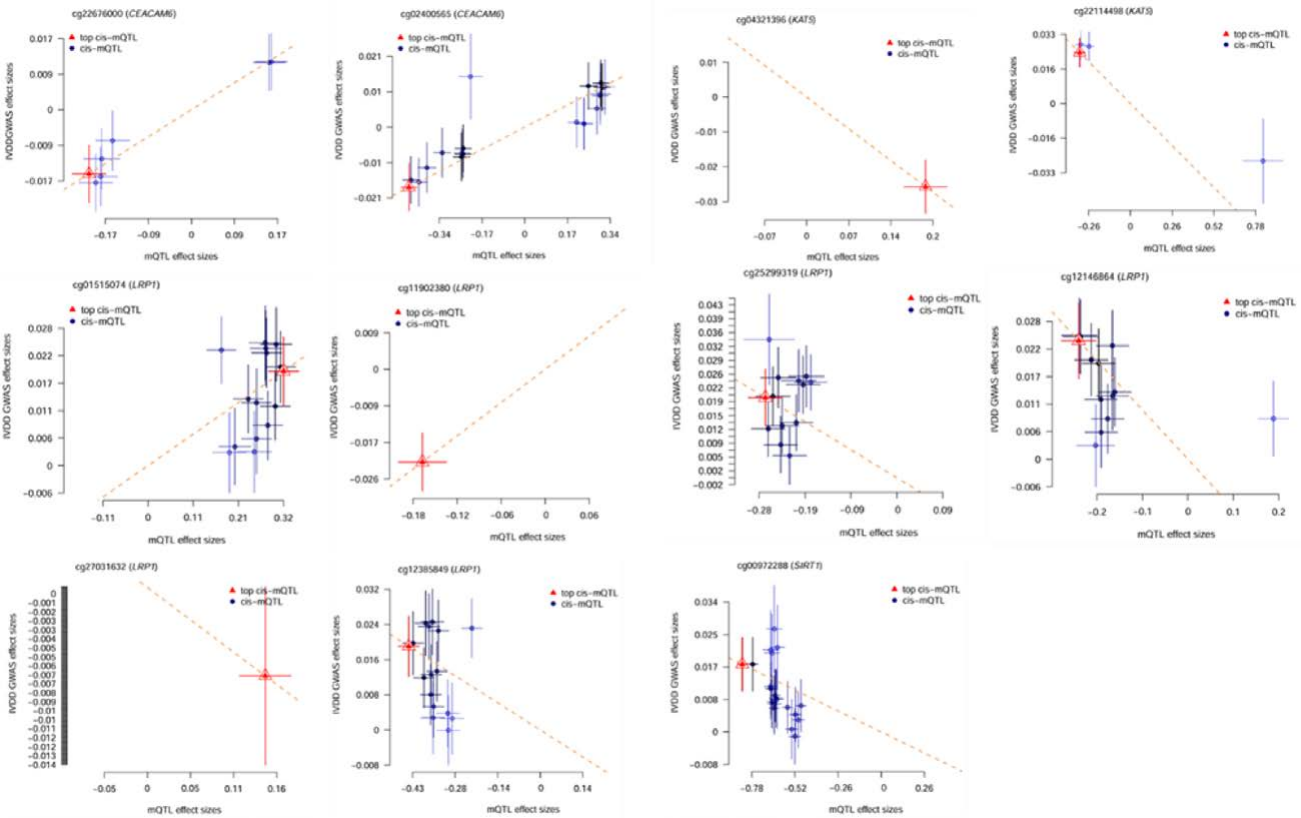
The effect of Lac-related genes methylation on IVDD. This figure shows the methylation effects of 11 CpG sites located near core genes (KAT5, SIRT1, LRP1, CEACAM6) that are associated with IVDD in the second step of SMR analysis (p_SMR < .05). Each subplot corresponds to 1 CpG site, with the horizontal axis representing mQTL effect sizes and the vertical axis representing GWAS effect sizes. The results are as follows: Negatively correlated sites (6): cg04321396 (KAT5), cg22114498 (KAT5), cg12385849 (LRP1), cg12146864 (LRP1), cg25299319 (LRP1), cg00972288 (SIRT1), suggesting that high methylation of these sites may reduce the risk of IVDD. Positively correlated sites (4): cg11902380 (LRP1), cg01515074 (LRP1), cg22676000 (CEACAM6), cg02400565 (CEACAM6). Among them, the positive correlation of cg01515074 (LRP1) is the most significant (p_SMR = .008), indicating that its high methylation may increase the risk of IVDD. Site with no significant association (1): cg27031632 (LRP1), whose methylation level has no statistical association with IVDD risk. IVDD = intervertebral disc degeneration.

Table 2 shows the results of the second step of SMR analysis, the effect of Lac-related genes methylation on IVDD. A total of 27 CpG sites were near 14 genes after removing p_HEIDI < .01, When overlapping these results with the causal genes identified at the expression level, 11 CpG sites (near 4 genes, namely KAT5, SIRT1, LRP1, CEACAM6,) were found to have consistent effects, suggesting a potential interplay between DNA methylation and gene expression in regulating IVDD risk (Fig. 3).

**Table 2:** The effect of Lac-related genes methylation on IVDD.

	probeID	Gene	topSNP	p_SMR	p_HEIDI
1	cg07324293	OSBP2	rs5753318	.001	.585
2	cg17941049	ATXN7	rs831691	.001	.103
3	cg16258503	ATXN7	rs6779372	.002	.130
4	cg10480329	CENPA	rs11692689	.002	.367
5	cg04321396	KAT5	rs11820062	.005	.062
6	cg22114498	KAT5	rs113218949	.034	.044
7	cg12385849	LRP1	rs1800154	.007	.014
8	cg12146864	LRP1	rs11172124	.007	.031
9	cg25299319	LRP1	rs1800174	.007	.012
10	cg01515074	LRP1	rs1800168	.008	.040
11	cg27031632	LRP1	rs55909821	.034	.151
12	cg11902380	LRP1	rs7487148	.025	.065
13	cg15722533	TEAD1	rs7940191	.009	.951
14	cg27188243	TEAD1	rs7927865	.015	.791
15	cg12399536	TEAD1	rs4757953	.015	.746
16	cg25037165	TEAD1	rs7931147	.036	.273
17	cg25843174	TEAD1	rs3890064	.042	.333
18	cg00972288	SIRT1	rs10823119	.011	.236
19	cg24318537	UNC119B	rs6490300	.014	.233
20	cg01269191	KRAS	rs7311692	.024	.064
21	cg19116814	GPM6A	rs6553899	.031	.514
22	cg20378687	KPNA4	rs4679892	.032	.118
23	cg05194552	CREBBP	rs2530890	.037	.582
24	cg00039463	CREBBP	rs11641279	.038	.053
25	cg22676000	CEACAM6	rs8105477	.042	.882
26	cg02400565	CEACAM6	rs11878239	.045	.925
27	cg25320780	SLC16A4	rs814326	.045	.699

**Figure 4.**
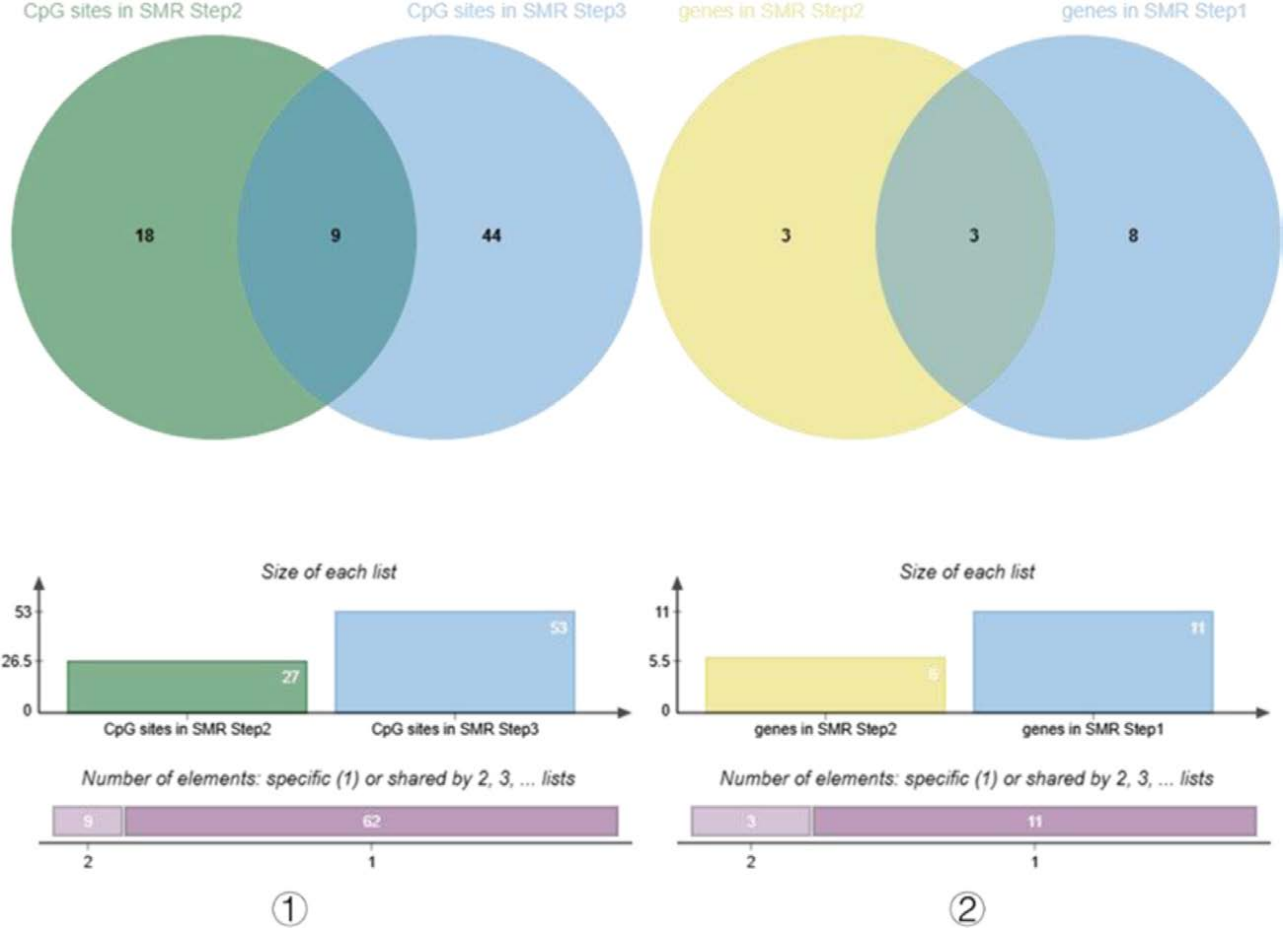
Venn diagram of intersecting CpG sites and intersecting genes. This figure shows the overlap of CpG sites and genes screened in different analysis steps through 3 overlapping circles: The first circle on the left: 27 significant CpG sites from the second step of SMR analysis (association between methylation and IVDD); The second circle on the left: 53 significant CpG sites from the third step of SMR analysis (association between methylation and gene expression); Left overlapping result: 9 significant CpG sites associated with both IVDD and gene expression. The first circle on the right: 6 significant genes from the second step of SMR analysis (association between methylation and IVDD); The second circle on the right: 11 significant genes from the first step of SMR analysis (association between gene expression and IVDD); Right overlapping result: 3 significant genes associated with both IVDD and methylation. IVDD = intervertebral disc degeneration.

**Figure 5.**
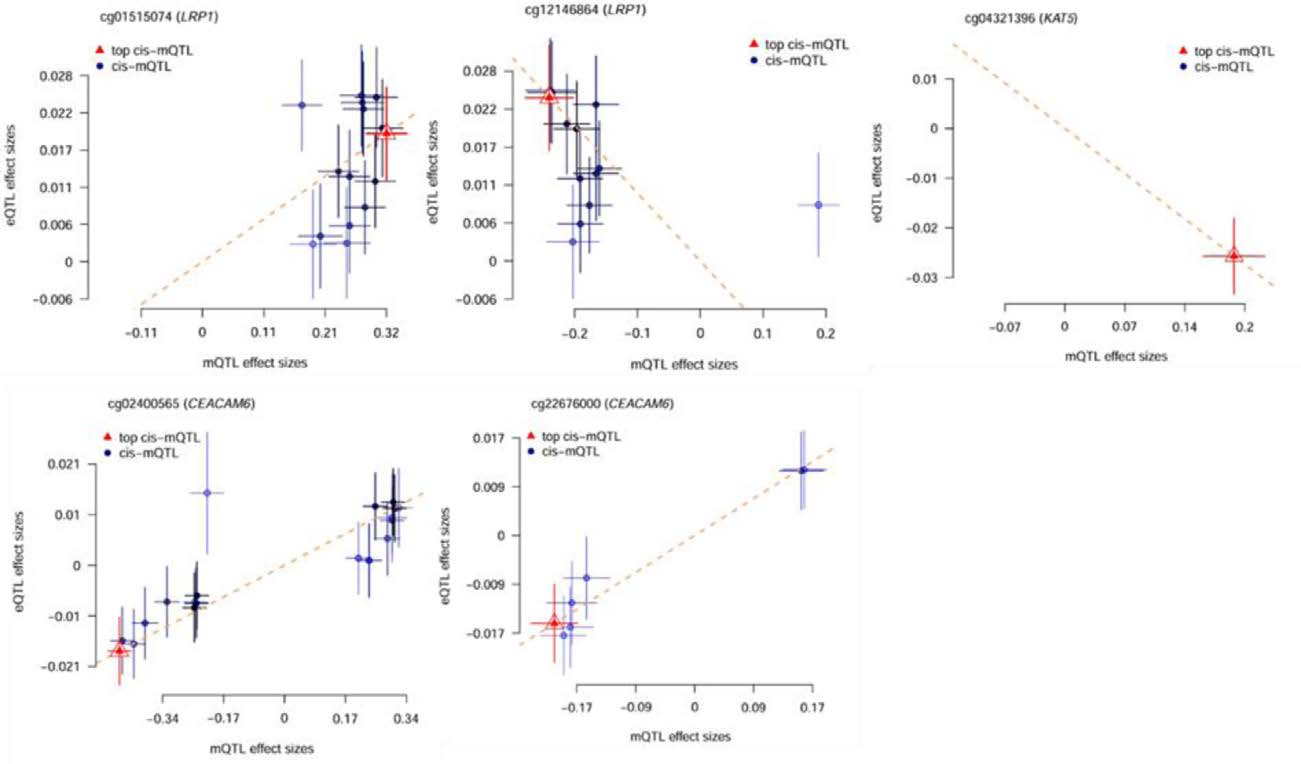
The effect of Lac-related genes methylation on lactate gene expression. This figure shows the regulatory effects of 5 core CpG sites on the expression of their target genes in the third step of SMR analysis (p_SMR < .05, p_HEIDI > .01). Each subplot corresponds to a CpG site-gene pair, with the horizontal axis representing mQTL effect sizes and the vertical axis representing eQTL effect sizes. The results are as follows: KAT5: Methylation of cg04321396 significantly inhibits KAT5 expression (negative effect size). CEACAM6: Methylation of both cg02400565 and cg22676000 significantly increases CEACAM6 expression (positive effect size). LRP1: Methylation of cg12146864 significantly inhibits LRP1 expression (negative effect size), while methylation of cg01515074 significantly increases LRP1 expression (positive effect size), showing differences in regulatory effects.

Table 3 shows the results of the third step of SMR analysis, the effect of Lac-related genes methylation on lactate gene expression. According to p_SMR < .05 and p_HEIDI > .01, a total of 53 CpG sites were expressed close to 27 genes, and after intersection analysis with the results of the second step of SMR analysis, a total of 9 CpG sites were obtained close to 6 genes, which were subsequently intersected with the results of the first step of SMR, and 5 CpG sites were obtained close to 3 genes after the visualization process (Fig. 4). Visualization of these 5 CpG sites revealed distinct regulatory patterns: methylation of the CpG site near KAT5 (cg04321396) inhibited KAT5 expression; methylation of the 2 CpG sites near CEACAM6 (cg02400565, cg22676000) promoted CEACAM6 expression; and methylation of the 2 CpG sites near LRP1 showed opposing effects – one (cg12146864) inhibited LRP1 expression, while the other (cg01515074) increased LRP1 expression (Fig. 5).

**Table 3:** The effect of Lac-related genes methylation on lactate gene expression.

	Expo_ID	Gene	Outco_ID	topSNP	p_SMR	p_HEIDI
1	cg27642470	DCBLD1	ENSG00000164465	rs2104064	.000	.012
2	cg07643096	DCBLD1	ENSG00000164465	rs13202514	.000	.054
3	cg27064063	DCBLD1	ENSG00000164465	rs138240393	.000	.027
4	cg03375056	YTHDF2	ENSG00000198492	rs4654320	.000	.275
5	cg03731740	YTHDF2	ENSG00000198492	rs3753692	.000	.061
6	cg11412288	DCBLD1	ENSG00000164465	rs7756187	.000	.012
7	cg24586444	DCBLD1	ENSG00000164465	rs3752848	.000	.017
8	cg22676000	CEACAM6	ENSG00000086548	rs8105477	.000	.821
9	cg02400565	CEACAM6	ENSG00000086548	rs11878239	.000	.013
10	cg20558659	OSBP2	ENSG00000184792	rs57527354	.000	.314
11	cg10139651	OSBP2	ENSG00000184792	rs139129417	.000	.415
12	cg16550651	KAT2A	ENSG00000108773	rs2285657	.000	.018
13	cg26916966	KAT2A	ENSG00000108773	rs731151	.000	.031
14	cg04321396	KAT5	ENSG00000172977	rs11820062	.000	.025
15	cg11637721	KAT5	ENSG00000172977	rs11227302	.001	.052
16	cg07388700	NMNAT1	ENSG00000173614	rs41280780	.000	.035
17	cg23610023	NMNAT1	ENSG00000173614	rs77972069	.001	.026
18	cg25664021	LRP1	ENSG00000182199	rs3024981	.008	.034
19	cg06715885	LRP1	ENSG00000182199	rs3024981	.009	.081
20	cg10233454	LRP1	ENSG00000182199	rs4759045	.010	.015
21	cg18477429	LRP1	ENSG00000182199	rs116842709	.033	.127
22	cg01515074	LRP1	ENSG00000123384	rs1800168	.001	.016
23	cg12146864	LRP1	ENSG00000123384	rs11172124	.000	.160
24	cg00693253	LRP1	ENSG00000123384	rs1800181	.000	.010
25	cg16258503	ATXN7	ENSG00000163635	rs6779372	.012	.066
26	cg17941049	ATXN7	ENSG00000163635	rs831691	.023	.016
27	cg21735376	YTHDF2	ENSG00000198492	rs185937751	.026	.373
28	cg20388635	YTHDF2	ENSG00000198492	rs185937751	.027	.379
29	cg10476962	TRAP1	ENSG00000005339	rs12929402	.046	.103
30	cg24062157	TRAP1	ENSG00000005339	rs12929402	.046	.115
31	cg04439028	TRAP1	ENSG00000126602	rs1053874	.000	.013
32	cg19116814	GPM6A	ENSG00000150625	rs6553899	.000	.074
33	cg10502220	GPM6A	ENSG00000150625	rs12498839	.000	.065
34	cg23678165	TFEB	ENSG00000112561	rs73733015	.003	.129
35	cg10696677	TFEB	ENSG00000112561	rs9462739	.000	.537
36	cg18076651	RARG	ENSG00000172819	rs117573579	.000	.387
37	cg13937905	RARG	ENSG00000172819	rs2272300	.049	.017
38	cg26462212	AXIN1	ENSG00000103126	rs387467	.000	.626
39	cg18337525	RUNX2	ENSG00000124813	rs7749637	.000	.248
40	cg04583149	STK11	ENSG00000118046	rs3764640	.000	.062
41	cg23387468	LUC7L2	ENSG00000146963	rs10085814	.000	.872
42	cg08691422	TP53	ENSG00000141510	rs12944939	.021	.029
43	cg00521887	PIK3C3	ENSG00000078142	rs3813065	.000	.224
44	cg15880760	CHAD	ENSG00000167107	rs9916635	.000	.119
45	cg19802458	FIS1	ENSG00000214253	rs10241107	.000	.958
46	cg18724891	UNC119B	ENSG00000175970	rs78321022	.000	.148
47	cg13023210	PAX6	ENSG00000007372	rs61879810	.003	.094
48	cg13636189	NR4A3	ENSG00000119508	rs10988896	.005	.296
49	cg23317857	TFEB	ENSG00000112561	rs60442837	.008	.032
50	cg13568622	ALDOB	ENSG00000136872	rs75061787	.000	.057
51	cg25320780	SLC16A4	ENSG00000168679	rs814326	.000	.143
52	cg02264195	TRAP1	ENSG00000126602	rs56026118	.000	.091
53	cg06625004	ARF1	ENSG00000143761	rs11582265	.000	.043

After integrating the results of multi-group SMR analyses, according to the mediator hypothesis model we constructed, SNPs affect traits by altering the level of Lac-DNAm, which in turn regulates the expression of lactylation functional genes, and ultimately has an effect on IVDD. kAT5 and CEACAM6 were excluded because the results of the 3-step analysis did not have consistency.

**Figure 6.**
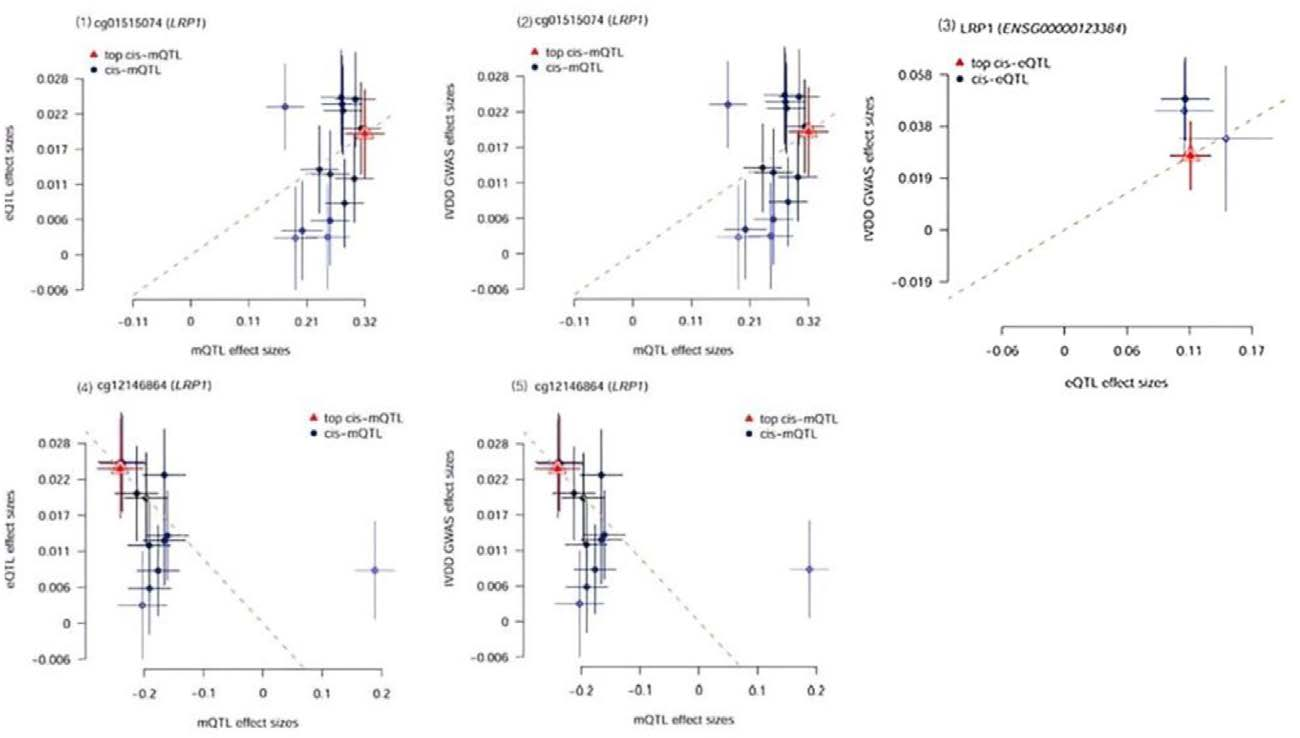
The effect of LRP1 methylation on LRP1 expression. This figure shows the cascade relationship among LRP1 methylation, expression, and IVDD risk through a series of subplots: Subplots (1) and (2): The methylation effect (mQTL) of cg01515074, showing that increased methylation level of cg01515074 can promote LRP1 expression (positive effect). Subplot (3): The association between LRP1 expression and IVDD risk (eQTL-GWAS), indicating that high LRP1 expression significantly increases IVDD risk (positive effect). Subplots (4) and (5): The methylation effect (mQTL) of cg12146864, showing that increased methylation level of cg12146864 can inhibit LRP1 expression (negative effect), but this effect is weaker than that of cg01515074 in the process of IVDD. Core conclusion: Methylation of cg01515074 upregulates LRP1 expression, while methylation of cg12146864 downregulates LRP1 expression, which finely regulates LRP1 expression. However, increased LRP1 expression ultimately increases the risk of IVDD. IVDD = intervertebral disc degeneration.

In this study, LRP1 emerged as the key gene with consistent causal effects. Specifically, methylation of cg01515074 was associated with increased LRP1 expression, while methylation of cg12146864 was linked to decreased LRP1 expression. Combined with the results of the first-step and second-step analyses, we found that the upregulation of LRP1 was ultimately associated with a higher risk of IVDD, confirming the role of high LRP1 expression in promoting IVDD (Fig. 6). In summary, the multi-omics SMR analysis identified that high LRP1 expression, as a critical feature of lactylation-related gene regulation in IVDD pathogenesis, is regulated by specific methylation sites (notably cg01515074 and cg12146864) and thereby influences disease risk.

## 6. Discussion

In this study, we used a multi-omics-based Mendelian randomization analysis to identify Lac-related genes as therapeutic targets for IVDD. Integrating pooled GWAS data on IVDD as well as mQTL and eQTL for Lac-related genes, our analysis prioritized high LRP1 expression and the cg01515074 and cg12146864 CpG loci. Our findings contribute to the understanding of genetic factors in IVDD under the lactylation pathway.

The IVD is a complex tissue.^[15]^ In normal healthy IVD, cell metabolism produces lactate, senescence occurs, and apoptosis, chronic damage to various structures of the IVD occurs as a function of stress, increasing pro-inflammatory factor expression and vascular infiltration, a variety of immune cells (e.g., macrophages) are induced to enter the IVD and participate in the regulation of IVDD.^[6]^ Past studies have confirmed^[4,16]^ that IVDD is in a state of chronic inflammation, increased multiple pro-inflammatory factors such as IL-1α, IL-1β and TNF-α, this results in the disc being in a state of metabolic imbalance, loss of extracellular matrix, and apoptotic cell death. In addition, vascular infiltration occurs after IVDD, and immune cells, such as macrophages, begin to enter the IVD and participate in changing the microenvironment of the IVD, which puts the IVDD in a complex dynamic process of degeneration and repair.^[6,17]^

Macrophages are functionally polarized and are usually divided into 2 subpopulations: classically activated macrophages (M1) and selectively activated macrophages (M2), with pro-inflammatory effects of M1 macrophages and anti-inflammatory effects of M2 macrophages.^[18,19]^ At the initial stage of macrophage entry into the IVDD, they polarize to M1 macrophages in response to inflammatory factors, increasing intradiscal inflammation.^[20]^ However, over time, macrophages undergo lactylation in response to lactate drivers, which manifests itself as conversion to M2 macrophages and reduced polarization to M1, ultimately exerting anti-inflammatory effects to regulate the IVD microenvironment and have a reparative effect on IVDD.^[21–23]^ In addition, the different polarization states of macrophages influence efferocytosis effects, with M1 macrophages exhibiting inhibition of efferocytosis in inflammatory states, whereas M2-types exhibit promotion of efferocytosis.^[24]^ Efferocytosis can remove apoptotic nucleus pulposus cells and other cells in the intervertebral disc, and enhanced efferocytosis can effectively delay IVDD, thus M2 macrophages are considered to be a discIVDD repair cell, and macrophage lactoylation plays an important role in IVDD.^[4]^

LRP1 is a low-density lipoprotein receptor-associated protein that is commonly expressed in the human body and is involved in the functional regulation of a variety of tissues and cells, mainly including neuronal cells, epithelial cells, muscle cells, fibroblasts, neuroglia, monocytes, macrophages, hepatocytes, adipocytes, vascular smooth muscle cells, and tumor cells, and is involved in energy metabolism, angiogenesis, neuronal homeostasis, and neurological degenerative lesions, etc.^[25,26]^ It was found that LRP1 inhibits lactylation by inhibiting lactate production from glycolysis.^[26]^ Our study found that when LRP1 was highly expressed, it increased the risk of IVDD, which may be related to the fact that high expression of LRP1 inhibits lactate production and thus affects lactylation, and when macrophage lactylation is inhibited, M2 macrophage transformation and efferocytosis are suppressed, which affects the repair of IVDD.

LRP1 gene expression is regulated by multiple CpG sites, and in this study, it was mainly considered to be significantly associated with cg01515074 and cg12146864. cg01515074-regulated LRP1 methylation can be associated with an increase in high LRP1 expression and an increased risk of IVDD, which is consistent with the negative impact of LRP1 gene expression on the risk of IVDD. In addition, we also found that methylation of cg12146864, which regulates LRP1 expression, is associated with a reduced risk of IVDD. Although cg12146864 and cg01515074 exhibit contradictory effects, this regulation reflects the fine-tuned epigenetic regulatory mechanism of LRP1 expression, which is critical for its role in IVDD pathogenesis. In the UCSC Genome Browser (https://genome.ucsc.edu/), we found that cg12146864 is located in exon 23 of LRP1, with its codon phase “start = 1, end = 1” placing it within a continuous coding sequence corresponding to a highly conserved functional domain. Methylation at this site may interfere with the transcriptional elongation of RNA polymerase or disrupt the recognition of splice signal sequences at both ends of the exon, leading to abnormal splicing or the production of truncated mRNA, ultimately inhibiting LRP1 expression and thereby reducing IVDD risk. In contrast, cg01515074 is localized to exon 44, with a codon phase of “start = 0, end = 1” indicating that it continues the reading frame of the previous exon without altering the translation phase. Methylation here may enhance the stability of this structure to reduce degradation or expose ribosomal binding sites to accelerate translation, thereby increasing LRP1 protein expression and contributing to a higher risk of IVDD. Such opposing regulatory patterns collectively reinforce LRP1 as a key driver in IVDD pathogenesis through precise epigenetic modulation. Therefore, for IVDD repair, it is crucial to continue to study LRP1 gene methylation.

The identification of LRP1 as a key lactylation-related gene driving IVDD carries substantial therapeutic implications. Given the consistent association between high LRP1 expression and increased IVDD risk, targeting LRP1 could represent a promising strategy for mitigating disease progression. Specifically, interventions aimed at modulating LRP1 expression through its regulatory CpG sites, such as cg01515074 and cg12146864, may offer precise avenues for therapeutic intervention. For instance, strategies that reduce the methylation of cg12146864 or inhibit the methylation-driven upregulation of LRP1 via cg01515074 could potentially lower LRP1 expression, thereby alleviating its inhibitory effect on lactylation. Restoring lactylation capacity in this context might enhance macrophage polarization toward the reparative M2 phenotype and promote efferocytosis, processes critical for resolving inflammation and facilitating disc repair. Additionally, direct inhibition of LRP1 function, perhaps through small-molecule antagonists or antibody-based therapies, could disrupt its role in suppressing glycolytic lactate production, thereby rebalancing the metabolic and inflammatory microenvironment within the intervertebral disc. Such approaches would need to be validated in preclinical models, but they highlight the potential of LRP1 as an actionable target for developing novel, mechanism-based treatments for IVDD.

Additionally, situating our findings within the broader landscape of established IVDD-related pathways can further illuminate the unique therapeutic potential of targeting LRP1. For instance, canonical pathways implicated in IVDD,^[27–29]^ such as those involving pro-inflammatory cytokines (e.g., IL-1β, TNF-α), extracellular matrix degradation (e.g., MMPs, aggrecanases), and oxidative stress, have long been focal points for therapeutic development. However, these pathways often target downstream inflammatory or degenerative processes, whereas LRP1-mediated regulation of lactylation represents a more upstream metabolic-epigenetic node. Unlike interventions aimed at neutralizing cytokines or inhibiting matrix-degrading enzymes—approaches that may only address secondary manifestations of IVDD – modulating LRP1 could tackle a root mechanism linking metabolic imbalance (lactate accumulation), epigenetic regulation (methylation-driven gene expression), and immune homeostasis (macrophage polarization).

Notably, cross-talk between LRP1 and these established pathways merits exploration. For example, LRP1’s inhibition of lactylation might exacerbate pro-inflammatory signaling by impairing M2 macrophage function, thereby amplifying cytokine-mediated damage – interaction that could explain why targeting LRP1 might concurrently dampen multiple degenerative cascades. Conversely, compared to pathways centered on aging-related senescence or mechanical stress, which are often difficult to reverse, the epigenetic regulation of LRP1 (via cg01515074 and cg12146864) offers a more druggable target, as epigenetic modifiers (e.g., DNA methyltransferases inhibitors) have shown promise in other disease contexts. Future research could explore combinatorial strategies: pairing LRP1 inhibition with anti-cytokine therapies (e.g., IL-1 receptor antagonists) to synergistically dampen inflammation, or combining epigenetic modulation of LRP1 with matrix-stabilizing agents (e.g., growth factors promoting aggrecan synthesis) to address both metabolic imbalance and structural deterioration. Such multi-target approaches, guided by LRP1 as a central regulator, could enhance treatment efficacy and reduce the risk of disease recurrence. Importantly, translational success will depend on bridging basic science with clinical needs – for example, refining delivery systems to penetrate the avascular disc environment, or addressing inter-individual variability in LRP1 regulation through pharmacogenomic profiling. By prioritizing these translational milestones, LRP1 could evolve from a mechanistic insight to a cornerstone of IVDD management, transforming both how we diagnose and treat this prevalent condition. By bridging metabolic, epigenetic, and immune processes, the LRP1-lactylation axis thus complements existing pathways, providing a novel, multi-faceted angle for therapeutic intervention in IVDD.

Although the SMR method is a well-established and robust approach for causal inference, this study has several limitations that should be acknowledged. First, the dataset used was predominantly from a European population, which may restrict the generalizability of our findings. Future studies should include more ethnic groups to enhance the applicability of the results across diverse populations. Second, regarding the selection of lactylation-related genes, while the GeneCards database integrates information from nearly 200 authoritative sources and offers comprehensive multi-omics data, our exclusive reliance on this database may introduce limitations. Different databases (e.g., STRING, OMIM) employ distinct criteria for defining “lactylation association”: for instance, some genes validated in experimental studies to interact with lactate metabolism or lactylation machinery might not be included in GeneCards due to its stricter inclusion thresholds. This could reduce the coverage of our gene set and limit the comprehensiveness of subsequent causal analyses, thereby narrowing the scope of the study. Third, our analysis was restricted to cis-eQTL and cis-mQTL data for lactylation-related genes, overlooking the potential role of trans-QTL. Trans-regulatory elements are known to participate in complex molecular networks underlying diseases. Their exclusion may have led to the omission of key regulatory pathways – such as long-range epigenetic interactions or cross-chromosomal genetic effects – that could influence the association between lactylation and IVDD, thereby hindering a complete understanding of the underlying mechanisms. Given these limitations, the relationship between IVDD, LRP1, and lactylation requires further analysis in larger, more diverse studies. Additionally, more experimental studies are necessary to functionally validate our findings.

## 7. Conclusion

Our study identified LRP1 as a lactylation-related gene that plays a key role in IVDD, providing new insights into the mechanisms and potential therapeutic targets of IVDD. The integration of genetic and epigenetic data is a powerful analytical approach that provides a research foundation for molecular studies of IVDD.

## Acknowledgments

Acknowledge anyone who contributed towards the article for authorship including anyone who provided professional writing services or materials.

## Author contributions

**Conceptualization:** Yang Yang, Xi Gao.

**Formal analysis:** Zhen Ai.

**Methodology:** Yang Yang.

**Resources:** Yang Yang.

**Software:** Yang Yang, Zhen Ai.

**Supervision:** Xi Gao.

**Validation:** Dingxuan Liu.

**Visualization:** Dingxuan Liu.

**Writing – original draft:** Yang Yang.

**Writing – review & editing:** Xi Gao.
